# Catalpol Protects ARPE-19 Cells against Oxidative Stress via Activation of the Keap1/Nrf2/ARE Pathway

**DOI:** 10.3390/cells10102635

**Published:** 2021-10-02

**Authors:** Longtai You, Hulinyue Peng, Jing Liu, Mengru Cai, Huimin Wu, Zhiqin Zhang, Jie Bai, Yu Yao, Xiaoxv Dong, Xingbin Yin, Jian Ni

**Affiliations:** School of Chinese Materia Medica, Beijing University of Chinese Medicine, Beijing 100029, China; 20190941307@bucm.edu.cn (L.Y.); 20200935154@bucm.edu.cn (H.P.); 20210941441@bucm.edu.cn (J.L.); cmrtcm@bucm.edu.cn (M.C.); 20190935128@bucm.edu.cn (H.W.); 20190935126@bucm.edu.cn (Z.Z.); 20190935127@bucm.edu.cn (J.B.); 20190935142@bucm.edu.cn (Y.Y.)

**Keywords:** catalpol, oxidative stress, Nrf2, apoptosis, cell cycle arrest, age-related macular degeneration

## Abstract

Oxidative damage to retinal pigment epithelial (RPE) has been identified as one of the major regulatory factors in the pathogenesis of age-related macular degeneration (AMD). Catalpol is an iridoid glucoside compound that has been found to possess potential antioxidant activity. In the present study, we aimed to investigate the protective effect of catalpol on RPE cells under oxidative stress and to elucidate the potential molecular mechanism involved. We found that catalpol significantly attenuated hydrogen peroxide (H_2_O_2_)-induced cytotoxicity, G0/G1 phase cell cycle arrest, and apoptosis in RPE cells. The overproduction of reactive oxygen species (ROS) and malondialdehyde (MDA) stimulated by oxidative stress and the corresponding reductions in antioxidant glutathione (GSH) and superoxide dismutase (SOD) levels were largely reversed by catalpol pretreatment. Moreover, catalpol pretreatment markedly activated the expression of nuclear factor (erythroid-derived 2)-like 2 (Nrf2) and its downstream antioxidant enzymes, catalase (CAT), heme oxygenase-1 (HO-1), and NADPH dehydrogenase (NQO1). It also increased the expression levels of cyclin E, Bcl-2, cyclin A, and cyclin-dependent kinase 2 (CDK2) and decreased the expression levels of Bax, Fas, cleaved PARP, p-p53, and p21 cleaved caspase-3, 8, and 9. The oxidative stress-induced formation of the Keap1/Nrf2 complex in the cytoplasm was significantly blocked by catalpol pretreatment. These results indicate that catalpol protected RPE cells from oxidative stress through a mechanism involving the activation of the Keap1/Nrf2/ARE pathways and the inactivation of oxidative stress-mediated pathways of apoptosis.

## 1. Introduction

Age-related macular degeneration (AMD) is an irreversible visual impairment associated disease prevalent in the elderly population around the world. The clinical symptoms of AMD are the visual distortion of straight lines, the presence of central dark spots, and decreased central vision [1,2,3,4,5]. Oxidative stress-induced retinal pigment epithelial (RPE) cell dysfunction and apoptosis have been identified as crucial factors involved in the pathogenesis of early AMD [6]. RPE cells are of highly active monolayer cells on the Bruch’s membrane between the neurosensory retina and the vascular choroid, and they play a very important role in maintaining the functions of retina and photoreceptors [7]. Therefore, RPE cells are vulnerable to the oxidative stress damage caused by stimulation from external factors (such as smoke and ultraviolet radiation) or high levels of intracellular reactive oxygen species (ROS) [8]. Oxidative stress damage leads to mitochondrial dysfunction and the production of abnormal lipid molecules in RPE cells. These products accumulate inside the Bruch’s membrane, leading to the formation of drusen which is one of the hallmarks of early AMD [9,10]. A growing corpus of evidence suggests that antioxidants, by inhibiting the production of ROS, protect RPE cells from oxidative stress, thereby playing a key therapeutic role in early AMD [11]. Therefore, investigations on the antioxidant pathways involved in retinopathy are crucial for the development of new therapies for AMD.

Nrf2-mediated activation of the antioxidant pathway is one of the key endogenous defense mechanisms for maintaining cellular redox balance [12]. Overproduction of intracellular ROS stimulates the dissociation of Nrf2 from Keap1 and its translocation to the nucleus, where Nrf2 binds to its downstream antioxidant response elements (AREs) to trigger the transcriptional activation of many phase II detoxifying and antioxidant enzymes, such as HO-1, glutaredoxin 1 (Grx1), CAT, and NQO1, thereby mitigating cellular redox imbalance caused by oxidative stress [13,14,15,16]. On the other hand, the uncontrolled and continuous generation of ROS further leads to DNA damage and mitochondrial dysfunction, thereby activating downstream caspase-dependent apoptotic pathway [17,18]. In addition, it was confirmed that activation of Nrf2 signaling pathway could inhibit ROS-mediated apoptosis in RPE cells [19]. Therefore, the development of novel antioxidant drugs targeting the activation of the Keap1/Nrf2/ARE antioxidant pathway is considered a promising approach to the treatment of AMD and other degenerative diseases involving a delicate intracellular oxidant–antioxidant imbalance.

Catalpol, an iridoid glucoside extracted from the dry roots of *Rehmannia glutinosa* Libosch, has been widely used to treat AMD and other degenerative diseases [20,21]. Recent studies indicate that catalpol exerts various biological properties, such as antioxidant, anti-apoptosis, neuroprotective, and anti-inflammation effects [22,23,24]. Although the mechanism involved in the treatment of certain diseases with catalpol has been gradually understood in the past few years, the potential of catalpol in the treatment of retinal diseases is still unknown. The purpose of this study was to investigate whether catalpol could protect RPE cells from oxidative stress damage and to further elucidate its role in the antioxidant defense mechanism. This study provides a novel therapeutic strategy for treating AMD or slowing down its progression.

## 2. Materials and Methods

### 2.1. Reagents and Chemicals

Catalpol (purity > 98:72%) was obtained from Shanghai Yuanye Bio-Technology Co., Ltd. (Shanghai, China). Fetal bovine serum (FBS), 0.05% trypsin, Dulbecco’s Modified Eagle Medium: Nutrient Mixture F-12 (DMEM/F-12), and penicillin/streptomycin solutions were purchased from Gibco, Invitrogen (Carlsbad, CA, USA). Dimethyl sulfoxide (DMSO), N-Acetylcysteine (NAC) and hydrogen peroxide (H_2_O_2_) were bought from Sigma-Aldrich (St. Louis, MO, USA). Phosphate-buffered saline (PBS) and 3-(4,5- dimethyl thiazol-2-yl-)-2,5-diphenyl tetrazolium bromide (MTT) were obtained from Beijing Solarbio Science and Technology Co. Ltd. (Beijing, China). ROS, MMP, LDH, TUNEL, Apoptosis and Cell Cycle detection kits were purchased from Beyotime Biotechnology (Shanghai, China).

### 2.2. Cell Cultures and Treatment

Human retinal pigment epithelial cells (ARPE-19) were purchased as frozen vials from BeNa Culture Collection (Beijing, China), and all cell experiments were performed between the third and fifth generations. The cells were cultured in DMEM/F-12 (supplemented with 10% FBS, 1% streptomycin/penicillin) at 37 °C in a humidified atmosphere containing with 95% air and 5% CO_2_. Cells at 80–90% confluence were selected for subculture and subsequent experimentation. For H_2_O_2_-induced apoptotic studies, the cells were cultured overnight in DMEM/F12 containing 2% FBS and then incubated in serum-free medium for 30 min, followed by exposure to H_2_O_2_ (400 μM) for 6 h. Catalpol was dissolved in DMSO and stored at 4 °C. Under the experimental conditions, the concentration of DMSO was maintained below 0.1%, since our previous studies revealed that this concentration does not cause cellular damage.

### 2.3. Cell Viability Assay and Morphology Examination

In the determination of the cytoprotective effect of catalpol on ARPE-19 cells, cell viability was measured using MTT assay. The cells were plated in 96-well plates at a density of 5 × 10^3^ cells/well overnight. After treatment with varying concentrations of catalpol (10–40 μM) for 24 h, H_2_O_2_ (400 μM) was added and treatment was continued for another 6 h. Untreated cells served as control. After incubation, the cells were treated with 100 μL MTT working solution (0.5 mg/mL in new culture medium) for 4 h at 37 °C. Thereafter, 150 μL DMSO was added to each well to dissolve the water-insoluble formazan crystals formed. Cell viability was evaluated by measuring absorbance of the formazan solutions at 570 nm using a microplate reader (Thermo, Multiskan, GO, USA). Morphological changes in ARPE-19 cells were observed and photographed under an inverted Olympus IX71 microscope.

### 2.4. LDH Cytotoxicity Assay

The release of lactate dehydrogenase (LDH) was determined using a LDH cytotoxicity assay kit. The ARPE-19 cells were inoculated into 96-well plates at a density of 1.5 × 10^4^ cells/well and incubated as indicated in Section 2.3. At the end of treatment, the cells were centrifuged at 1000 rpm for 5 min to obtain supernatants. According to the manufacturer’s instructions, 100 μL of each supernatant was used to determine the content of LDH. The absorbance of the sample was determined on a Thermo microplate reader (Multiskan, GO, USA) at 490 nm with dual wavelength, using 600 nm as reference wavelength.

### 2.5. TUNEL Staining

One of the recognized marker events of apoptosis is DNA fragmentation. The specific DNA fragments can be measured using a terminal deoxynucleotidyl transferase (TdT) dUTP nick end labeling (TUNEL) assay. The ARPE-19 cells were seeded on coverslips at a density of 2 × 10^5^ cells/well and treated as indicated in Section 2.3. Thereafter, the cells were fixed with 4% (*w/v*) paraformaldehyde. Then, apoptosis was determined using TUNEL assay kit according to the manufacturer’s instructions. Apoptotic cells were identified and photographed using a florescence microscope (BX51-DSU; Olympus, Tokyo, Japan) at × 200 magnification.

### 2.6. Apoptosis Assay

The ARPE-19 cells were seeded in 6-well plates at a density of 2 × 10^5^ cells/well and cultured overnight. The cells were then incubated with either catalpol (10–40 μM) for 24 h, or NAC (10 mM) for 2 h before treatment with 400 μM H_2_O_2_ for 6 h. In brief, the cells were collected and stained in 300 μL of binding buffer containing 5 μL Annexin V–fluorescein isothiocyanate (FITC) and 10 μL propidium iodide (PI) for 25 min at room temperature in the dark. The stained cells were subjected to apoptosis analysis using a FACSCanto II flow cytometer (BD Biosciences, NJ, USA).

### 2.7. Measurement of ROS Levels

The levels of intracellular ROS were determined with 2,7-dichlorofluorescin diacetate (DCFH-DA) staining assay. The ARPE-19 cells were plated in 6-well plates at a density of 2 × 10^5^ cells/well and incubated as stated in Section 2.3. Thereafter, the cells were harvested and incubated with 10 mM DCFH-DA working solution using ROS assay kit at 37 °C for 30 min in the dark. The cells were washed and re-suspended in PBS, followed by flow cytometric analysis using flow cytometer to determine the percentage of fluorescence-positive cells.

### 2.8. Determination of the Oxidative Stress Index

As in previous sections, ARPE-19 cells were seeded and treated as described in Section 2.3. Then, the cells were lysed with ice-cold radio-immunoprecipitation assay (RIPA) buffer (Sigma, St. Louis, MO, USA). The total protein concentrations of the lysate supernatants were determined using the Bradford protein assay kit (Boster Biological Technology Co. Ltd., Wuhan, China). The levels of malondialdehyde (MDA), glutathione peroxidase (GSH-Px), catalase (CAT), glutathione (GSH), superoxide dismutase 2 (SOD2), and superoxide dismutase (SOD) in the supernatants were determined using their corresponding assay kits (Nanjing Jiancheng Bioengineering Institute, Nanjing, China) according to the manufacturers’ specifications. Data were calculated in terms of protein concentration of each sample.

### 2.9. Measurement of Mitochondrial Membrane Potential (Δψm)

Loss of mitochondrial membrane potential (MMP) was measured using an ideal fluorescent probe 5,5′,6,6′-tetrachloro-1,1′,3,3′-tetraethylbenzimidazolylcarbocyanine iodide (JC-1), which aggregates as a polymer (red fluorescence) in an undamaged mitochondrial matrix. A decrease in MMP promotes the dissociation of JC-1 into the cytoplasm and its existence as a monomer (green fluorescence) [25]. In normal cells, JC-1 enters the mitochondria through the mitochondrial membrane polarity and forms a multimer that emits red fluorescence due to the increased concentration, which is usually the FL-2 channel (same channel as PE) when detected by flow cytometry. When apoptosis occurs, the mitochondrial transmembrane potential is depolarized and JC-1 is released from the mitochondria and exists in the cytoplasm as a monomer emitting green fluorescence, which is usually the FL-1 channel (same channel as FITC) when detected by flow cytometry. Therefore, the change in mitochondrial membrane potential was detected by calculating the ratio of red/green (PE/FITC channel) fluorescence intensity in each group of cell population. In this study, the cells (ARPE-19) were seeded and treated as stated in Section 2.3. The cells were collected and resuspended in 1 mL of JC-1 working solution (10 μM) and incubated for 30 min at 37 °C in the dark. Then, the stained cells were washed twice with PBS and changes in intracellular MMP were measured using flow cytometry. Cell fragments or dead cells were appropriately gated using an FSC/SSC plot when detected by flow cytometry.

### 2.10. Cell Cycle Assay

The effect of catalpol on the cell cycle in ARPE-19 cells was determined with flow cytometry. The cells were seeded and treated as described in Section 2.3. At the end of incubation, the cells were collected, washed with PBS, and fixed in ice-cold 70% ethyl alcohol at 4 °C overnight. In this experiment, a cell cycle detection kit was used to measure the distribution of ARPE-19 cells in line with the kit manufacturer’s instructions. The fixed cells were washed twice with PBS and incubated with a working solution of PI/RNase A in the dark at 37 °C for 30 min. The samples were subjected to flow cytometric analysis in a FACSCanto II flow cytometer.

### 2.11. Western Blot Analysis

The ARPE-19 cells were treated as stated in Section 2.3, after which they were collected and washed twice with cold PBS. Total protein was extracted using ice-cold RIPA cell lysis buffer containing 1 mM phenylmethylsulfonyl fluoride (PMSF, Boster Biological Technology Co. Ltd., Wuhan, China). The protein concentration of each lysate was measured using the Bradford protein assay kit. A ProteoExtract^®^ Cytosol/Mitochondria Fractionation kit (Millipore, Billerica, MA, USA) was used to separate the mitochondrial and cytoplasmic proteins from lysed cells. Nucleoprotein was extracted with Nuclear and Cytoplasmic Protein Extraction Kit (Sangon Biotech Co. Ltd., Shanghai, China). Equal amounts of protein samples were separated on a 10% SDS- polyacrylamide gel electrophoresis and transferred to PVDF membrane (Millipore, Bedford, MA, USA). The membranes were blocked with TBST buffer containing 5% nonfat dried milk for 1 h, prior to incubation overnight at 4 °C with primary antibodies (Table 1). This was followed by incubation with appropriate secondary antibodies for 60 min at room temperature. Histone H3, COX IV, and β-actin were used as loading controls for nucleoprotein, mitochondrial protein, and total protein, respectively. The target protein bands were measured using Western Lightning^TM^ Chemiluminescence Reagent (PerkinElmer, Waltham, MA, USA) and analyzed with Labworks^TM^ 4.0 digital quantification software (UVP, USA).

### 2.12. Co-Immunoprecipitation (Co-IP) Assay

The cells were collected and lysed with ice-cold RIPA buffer containing 2% protease inhibitor cocktail (Sigma-Aldrich, St. Louis, MO, USA). A 50% slurry (10 μL) of protein A/G magnetic beads was added to the whole cell, followed by pre-incubation overnight at 4 °C. Anti-Keap1 antibody (2 μg) was added to the whole cell lysate and incubated overnight at 4 °C. The pretreated beads were added to the cell lysate (incubated overnight with Keap1 antibody) and incubated at 4 °C overnight. Immunoprecipitated complexes were washed with RIPA buffer and then boiled in 2× SDS loading buffer for 5 min. The presence and expression of the target protein Nrf2 in the supernatant were determined with immunoblotting.

### 2.13. Quantitative Real-Time Polymerase Chain Reaction (qRT-PCR) Analysis

The methods for total RNA extraction and qRT-PCR have been previously described [26]. In essence, ARPE-19 cells were treated as described in Section 2.3. Then, the cells were collected and the total RNA was isolated using TRIzol reagent (Invitrogen, Waltham, CA, USA). The following primer sequences were used: β-actin: forward: 5′-CCTAAGGCCAACCGTGAAA-3′; reverse: 5′-TGGTACGACCAGAGGCATA-3′; NQO1: forward: 5′- GAGGTGGTGGAGTCG-3′; reverse: 5′- GGATACTGAAAGTTCGC-3′. The quantitative expression of the NQO1genes was normalized to that of β-actin, and the mRNA expressions were calculated using the relative quantification method of 2^−ΔΔCt^.

### 2.14. Statistical Analysis

The GraphPad Prism 8.0 statistical software (La Jolla, CA, USA) was used for data analysis. Data are expressed as mean ± S.D. for a minimum of three independent experiments. One-way ANOVA and the LSD test were used to analyze significant differences in multiple and single comparisons, respectively. Values of *p* < 0.05 were considered statistically significant.

## 3. Results

### 3.1. Catalpol Inhibited H_2_O_2_-Induced Cytotoxicity in ARPE-19 Cells

**Figure 1:**
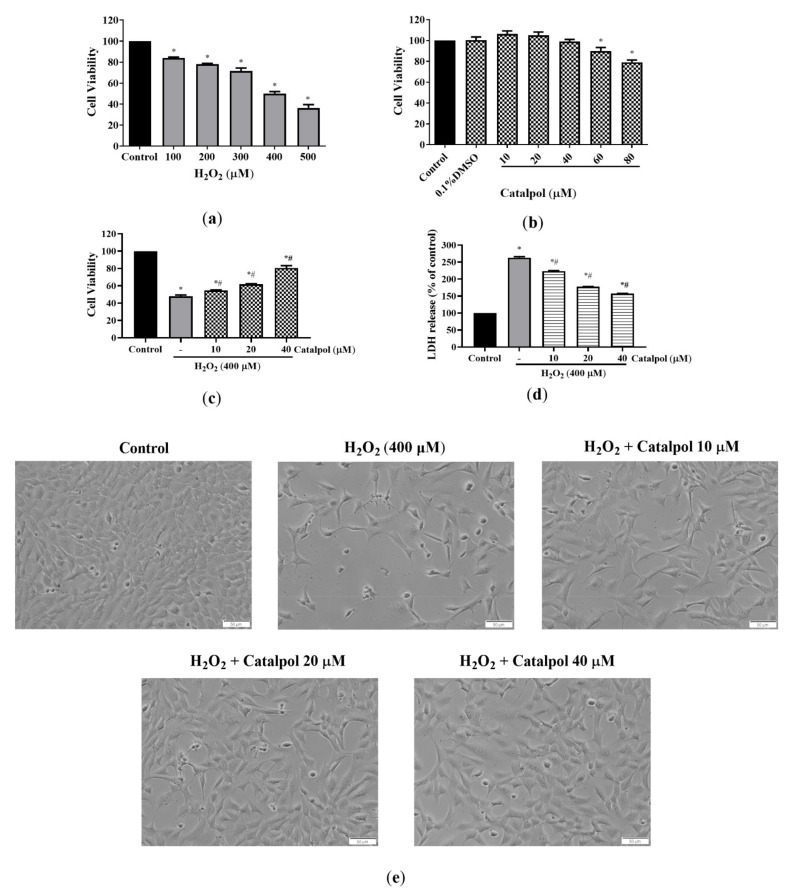
Catalpol protected RPE cells from H_2_O_2_-induced cell damage. (**a**) ARPE-19 cells were first exposed to different concentrations of H_2_O_2_ (100, 200, 300, 400, and 500 μM) for 6 h, and cell viability was determined with MTT assay. (**b**) Effect of different concentrations of catalpol on ARPE-19 cell viability. (**c**) Cytoprotective effect of catalpol. ARPE-19 cells were pretreated with varying concentrations of catalpol (10, 20, and 40 μM) for 24 h, and then H_2_O_2_ (400 μM) was added and the treatment was continued for 6 h. Cell viability was measured with MTT assay. (**d**) ARPE-19 cells were pretreated with catalpol (10–40 μM) for 24 h before being exposed to H_2_O_2_ (400 μM) for 6 h. The release of LDH was measured using an LDH cytotoxicity assay kit. (**e**) Changes in the number of ARPE-19 cells were observed and presented (original magnification: 200×). Data are presented as mean ± S.D. of three independent experiments (* *p* < 0.05 vs. control group, # *p* < 0.05 vs. H_2_O_2_ (400 μM)-treated group).

To determine the toxic effect of H_2_O_2_ on ARPE-19 cells, the cells were incubated with different concentrations of H_2_O_2_ (100–500 μM) for 6 h, and their viability was determined using MTT assay. As shown in Figure 1a, H_2_O_2_ significantly inhibited the viability of ARPE-19 cells in a dose-dependent manner. The IC_50_ value of H_2_O_2_ in ARPE-19 cells at 6 h was 400 μM, which was used in subsequent experiments. As shown in Figure 1b, 60 μM catalpol was cytotoxic to RPE cells. Thus, we used catalpol concentrations of 10, 20, and 40 μM in subsequent experiments. To evaluate the cytoprotective effect of catalpol, the cells were pretreated with increasing concentrations of catalpol (10–40 μM) for 24 h before exposure to 400 μM H_2_O_2_ for 6 h. Compared with the H_2_O_2_-treated group, catalpol pretreatment increased the viability of ARPE-19 cells in a dose-dependent manner (Figure 1c). The leakage of LDH is considered to be one of the essential events in the destruction of cell membrane integrity. This study observed that H_2_O_2_ treatment stimulated the release of LDH from ARPE-19 cells. However, pretreatment with catalpol at doses of 10–40 μM effectively prevented the release of LDH (Figure 1d). Moreover, the protective effect of catalpol on H_2_O_2_-treated ARPE-19 cells was further confirmed by observing changes in cell number (Figure 1e).

### 3.2. Catalpol Inhibited H_2_O_2_-Induced ARPE-19 Cell Apoptosis

**Figure 2:**
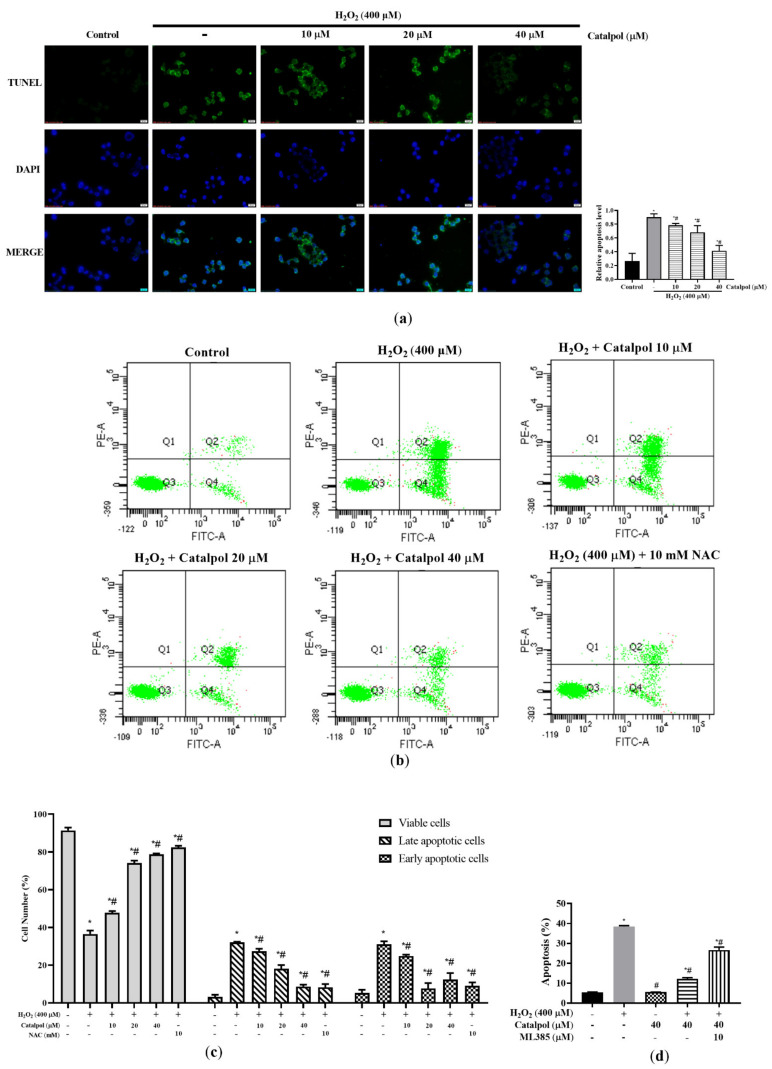
Catalpol exerted anti-apoptotic protective effect on H_2_O_2_-induced RPE cells. (**a**) ARPE-19 cells were treated with H_2_O_2_ (400 μM) in the presence or absence of catalpol (10, 20 and 40 μM) for 6 h. TUNEL assay was preformed according to the manufacturer’s instructions. The apoptotic cells were identified and captured under fluorescence microscopy (scale bar = 10 μm). (**b**,**c**). The cells were incubated with either catalpol (10–40 μM, 24 h) or NAC (10 mM, 2 h) for 24 h before being treated with 400 μM H_2_O_2_ for 6 h. Cell apoptosis was measured with Annexin V–FITC assay. (**d**) Pretreatment with ML385, an Nrf2 inhibitor, blocked catalpol-mediated reduction in apoptosis in H_2_O_2_-treated ARPE-19 cells. Data are presented as mean ± S.D. of three independent experiments (* *p* < 0.05 vs. control group, # *p* < 0.05 vs. H_2_O_2_ (400 μM)-treated group).

To investigate whether the cytoprotective effect of catalpol was involved in the regulation of apoptosis, cell apoptosis was determined with TUNEL nuclei staining and Annexin V-FITC/PI flow cytometry. As shown in Figure 2a, the percentage of apoptosis of TUNEL-positive cells due to H_2_O_2_ treatment was significantly increased. In contrast, catalpol pretreatment at doses of 10–40 μM inhibited H_2_O_2_-induced apoptosis. The anti-apoptotic effect of catalpol on in ARPE-19 cells was further verified using Annexin V-FITC/PI double staining (Figure 2b,c). Compared with the control group, the percentage of early and late apoptotic cells in the H_2_O_2_-treated group increased to 31.10 ± 1.53 and 32.10 ± 0.30%, respectively. However, pretreatment with catalpol at doses of 10–40 μM markedly reduced the degree of apoptosis of ARPE-19 cells treated with H_2_O_2_. Pretreatment with the ROS inhibitor N-acetylcysteine (NAC) (10 mM) effectively blocked H_2_O_2_-induced apoptosis in ARPE-19 cells (Figure 2c). Overall, these results suggest that the inhibition of apoptosis played a key role in the protective effect of catalpol on ARPE-19 cells.

### 3.3. Effect of Catalpol on H_2_O_2_-Induced Cell Cycle Arrest in ARPE-19 Cells

**Figure 3:**
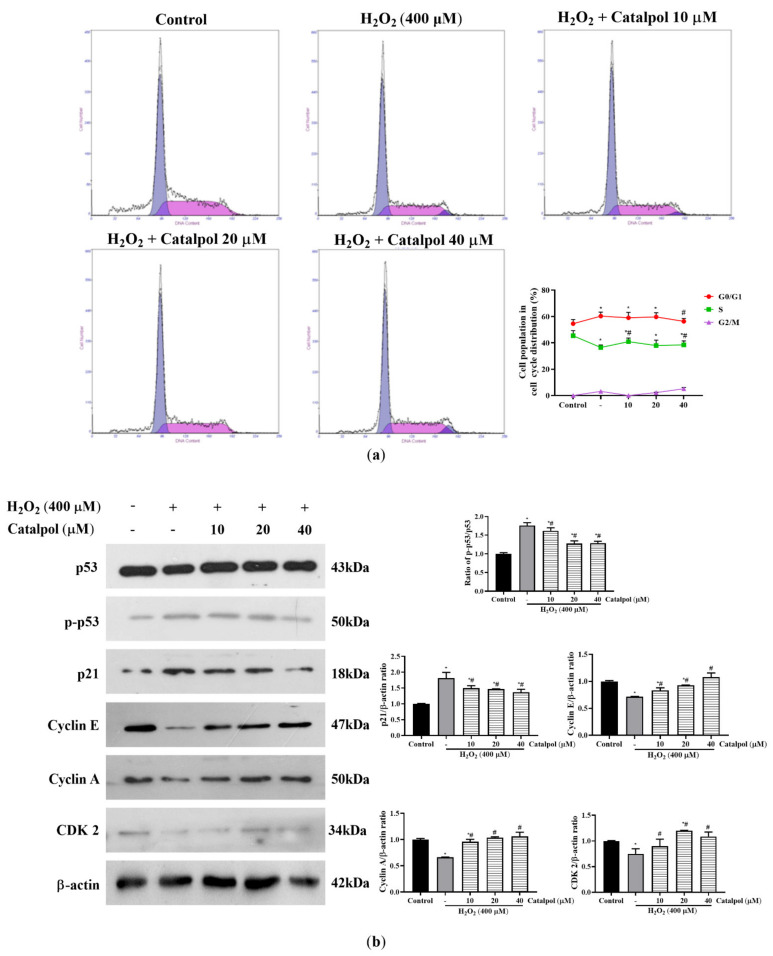
Effect of catalpol on cell cycle arrest in H_2_O_2_-induced RPE cells. (**a**) ARPE-19 cells were pretreated with or without catalpol (10, 20, and 40 μM) for 24 h and then treated with 400 μM H_2_O_2_ for 6 h. The cell cycle progression was measured using PI assay. (**b**) The expression levels of related regulatory proteins in the cell cycle were determined with Western blot assay. β-actin was used as internal control. Data are presented as mean ± S.D. of three independent experiments (* *p* < 0.05 vs. control group, # *p* < 0.05 vs. H_2_O_2_ (400 μM)-treated group).

Apoptosis is usually accompanied by cell cycle arrest. Therefore, to determine whether catalpol exerts its cytoprotective effect by regulating the cell cycle progression, the effects of catalpol on the cell cycle distribution of ARPE-19 cells were analyzed by PI staining and flow cytometry. Compared with untreated cells, it was observed that the S phase cells were significantly decreased in the H_2_O_2_-treated group, while the corresponding G0/G1 phase cells were significantly increased, indicating that H_2_O_2_ induced cell cycle arrest in the G0/G1 phase (Figure 3a). In contrast, catalpol pretreatment significantly mitigated H_2_O_2_-induced G0/G1 phase cell cycle arrest. Moreover, the results shown in Figure 3b suggest that catalpol pretreatment significantly blocked the H_2_O_2_-stimulated upregulations of protein expressions of p-p53, and p21 in ARPE-19 cells. The binding and activation of cyclins and cycle-dependent kinases (CDKs) are amongst the essential mechanisms that regulate the cell cycle [27]. As shown in Figure 3b, catalpol pretreatment effectively reversed the H_2_O_2_-mediated decreases in the expression of cyclin E, cyclin A, and CDK2 in ARPE-19 cells. Together, these results indicate that the regulation of cell cycle arrest was involved in the protective effect of catalpol on ARPE-19 cells.

### 3.4. Effect of Catalpol on the Modulation of Apoptosis-Related Proteins in H_2_O_2_-Treated ARPE-19 Cells

The apoptotic signaling cascade is dependent on the intrinsic or extrinsic apoptotic pathways that are activated by mitochondrial stimulation or molecules that bind to cell membrane death receptors, respectively [28]. Although both pathways are triggered by different stimuli, they directly activate downstream caspases, which are a series of cysteine proteases that regulate apoptosis activation [29].

**Figure 4:**
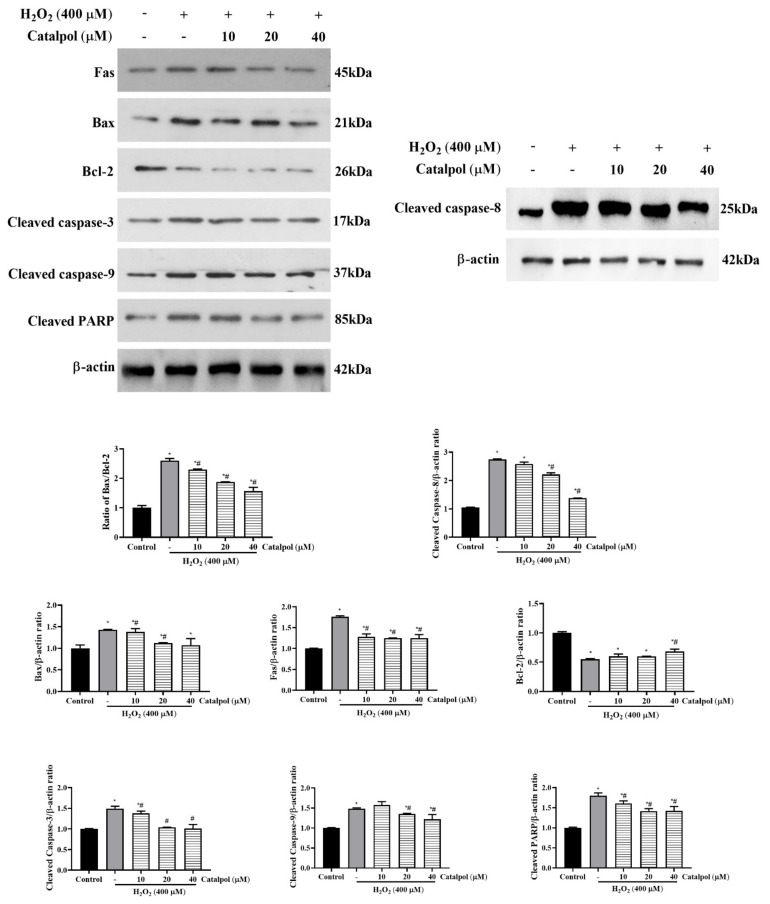
Regulation of apoptosis-related protein expression in H_2_O_2_-induced RPE cells by catalpol pretreatment. ARPE-19 cells were pretreated with or without catalpol (10, 20, and 40 μM) for 24 h and then treated with 400 μM H_2_O_2_ for 6 h. The bands and relative protein expressions of Bcl-2, Bax, Fas, cleaved caspase-3, cleaved caspase-8, cleaved caspase-9, and cleaved caspase-PARP were measured with Western blot assay. β-actin was used as internal control. Data are presented as mean ± S.D. of three independent experiments (* *p* < 0.05 vs. control group, # *p* < 0.05 vs. H_2_O_2_ (400 μM)-treated group).

Previous studies have shown that oxidative stress-induced mitochondrial dysfunction in RPE cells and activation of its downstream apoptotic pathway have been identified as key factors in the pathogenesis of early AMD [6,7,8,9,10]; therefore, we further investigated the mechanism of the anti-apoptotic effect of catalpol under oxidative stress. As shown in Figure 4, the results of WB suggest that the expression of cleaved caspase-9, cleaved caspase-3, and cleaved PARP were significantly upregulated in H_2_O_2_-treated ARPE-19 cells, when compared with the control group, whereas the expressions of these proteins were markedly down-regulated by catalpol pretreatment under oxidative stress. Caspase-8 is a critical apical activator caspase in the extrinsic apoptotic pathway, and its initiation is thought to be in response to the activation of upstream Fas protein (a typical death receptor) [30]. Exposure of the ARPE-19 cells to H_2_O_2_ (400 μM) for 6 h led to significant increases in the protein expressions of Fas and downstream cleaved caspase-8 (Figure 4). These were dose-dependently reversed by pretreatment with catalpol (10–40 μM). Collectively, these results suggest that catalpol inhibits apoptosis of ARPE-19 cells exposed to H_2_O_2_-induced oxidative stress by suppressing the activation of mitochondria-dependent and death receptor-mediated apoptotic pathways.

### 3.5. Catalpol Suppressed H_2_O_2_-Induced Oxidative Stress Activation in ARPE-19 Cells

**Figure 5:**
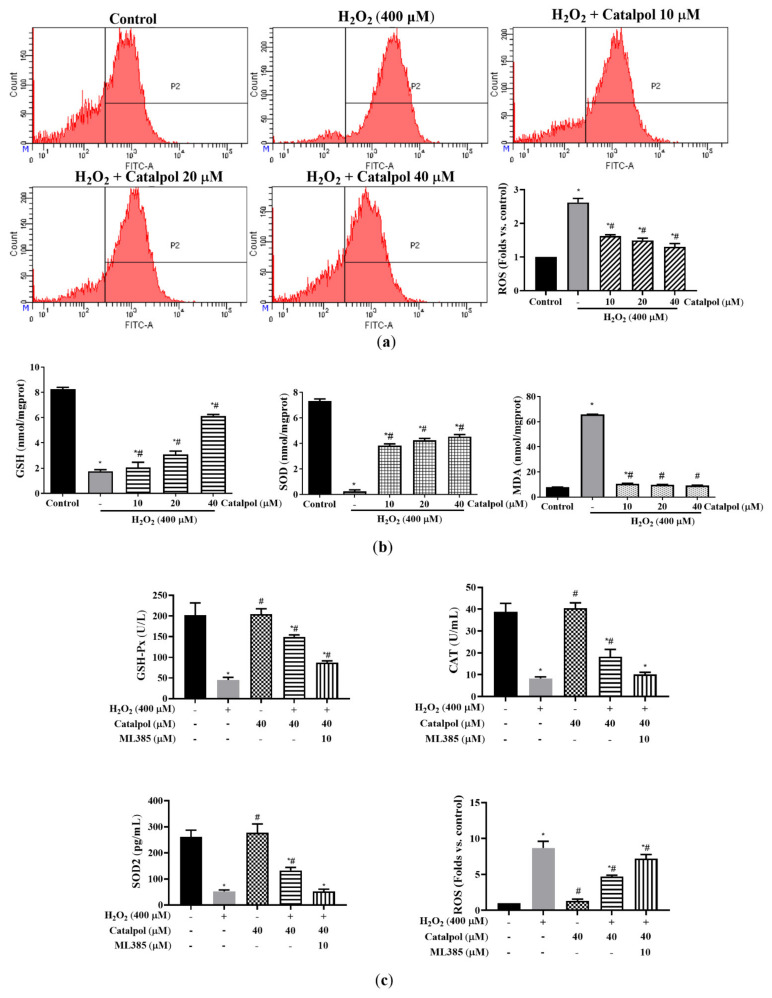
Effect of catalpol on ROS production and antioxidant enzyme activity in H_2_O_2_-treated RPE cells. (**a**) ARPE-19 cells were pretreated with or without catalpol (10, 20, and 40 μM) for 24 h and then treated with 400 μM H_2_O_2_ for 6 h. ROS production was measured and analyzed using the DCFH-DA assay. (**b**) The levels of cellular GSH, SOD, and MDA were determined. (**c**) Treatment with ML385, an Nrf2 inhibitor, blocked the antioxidant protective effects of catalpol on H_2_O_2_-treated ARPE-19 cells. The levels of cellular SOD2, CAT, GSH-Px, and ROS were determined. Data are presented as mean ± S.D. of three independent experiments (* *p* < 0.05 vs. control group, # *p* < 0.05 vs. H_2_O_2_ (400 μM)-treated group).

Overproduction of ROS is one of the critical indicators of oxidative stress that triggers apoptotic signals [31]. In order to determine whether the cytoprotective effect of catalpol in ARPE-19 cells was related to its antioxidant property, the cells were incubated with 400 µM H_2_O_2_ for 6 h in the presence or absence of catalpol (10–40 µM). As shown in Figure 5a,b, H_2_O_2_ treatment led to significant increases in intracellular ROS and MDA levels, which were effectively reversed by catalpol pretreatment. Glutathione (GSH), glutathione peroxidase (GSH-Px), superoxide dismutase 2 (SOD2), catalase (CAT), and SOD are natural antioxidative agents and enzymes in cells, and they are important for maintaining cellular redox balance [32,33]. The results shown in Figure 5b,c indicate that catalpol pretreatment significantly inhibited the reductions in GSH level and SOD, SOD2, CAT, and GSH-Px activities in H_2_O_2_-induced ARPE-19 cells. Moreover, we tried to confirm if intracellular ROS generation was involved in the initiation of oxidative stress-mediated apoptosis. It was found that pretreatment with NAC (a scavenger of ROS) significantly inhibited H_2_O_2_-induced apoptosis of ARPE-19 cells (Figure 2c). These results suggest that the protective effect of catalpol on ARPE-19 cells involved the inhibition of oxidative stress-mediated apoptosis.

**Figure 6:**
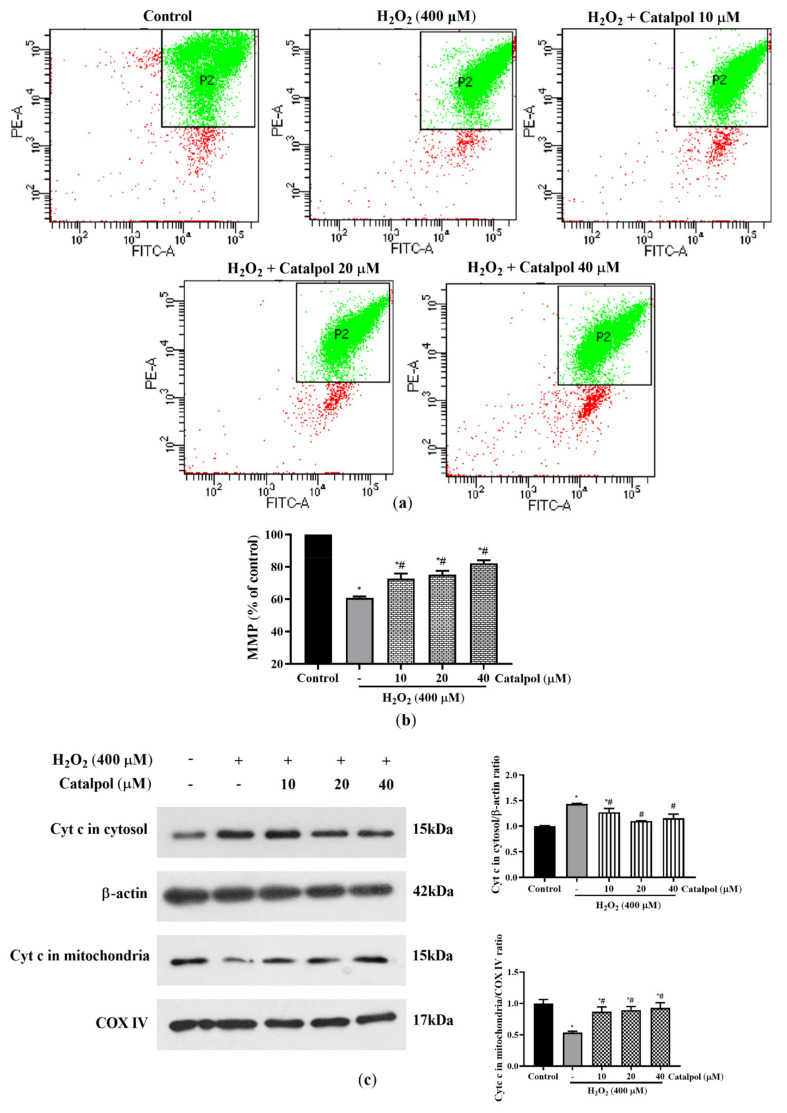
Effect of catalpol on mitochondrial dysfunction in H_2_O_2_-treated RPE cells. (**a** and **b**) ARPE-19 cells were pretreated with or without catalpol (10, 20, and 40 μM) for 24 h and then treated with 400 μM H_2_O_2_ for 6 h. Changes in intracellular MMP were measured and analyzed with the JC-1 assay. The fluorescence intensity ratio of PE/FITC channels within the P2 gate was expressed as a change in MMP. In addition, to demonstrate the differences between the treatment and control groups in a more visual manner, we normalized the corresponding PE/FITC fluorescence intensity ratio of the treatment group relative to the control group. (**c**) Cytochrome c levels in the mitochondria and cytosol were measured using Western blot. COX IV was used as internal control of the mitochondrial fraction, and β-actin was used as internal control for the cytosolic fraction. Data are presented as mean ± S.D. of three independent experiments (* *p* < 0.05 vs. control group, # *p* < 0.05 vs. H_2_O_2_ (400 μM)-treated group).

Excessive oxidative stress leads to mitochondrial damage and dysfunction, which are accompanied by abnormal production of ROS and the loss of MMP. The complete collapse of MMP promotes the release of cytochrome *c* from the mitochondria into the cytoplasm, thereby activating irreversible cell apoptosis [34,35]. The results in Figure 6a,b demonstrate that H_2_O_2_ treatment resulted in a significant loss of MMP in ARPE-19 cells, when compared to the control group. However, these changes were markedly mitigated by catalpol pretreatment at doses of 10–40 μM for 24 h pretreatment.

Bax and Bcl-2 are a pair of apoptosis-related proteins with opposite effects. They regulate the release of cytochrome c, thereby controlling mitochondria dependent apoptotic cell death [36]. As demonstrated in Figure 4, treatment of ARPE-19 cells with H_2_O_2_ markedly increased the ratio of Bax/Bcl-2. However, the expression levels of Bax and Bcl-2 were significantly reversed by pretreatment with 10–40 μM catalpol (Figure 4). Furthermore, compared with the H_2_O_2_-treated cells, it was found that catalpol pretreatment blocked the release of cytochrome *c* from the mitochondria into the cytoplasm (Figure 6c). Overall, these results suggest that the anti-apoptotic effect of catalpol on ARPE-19 cells may be involved in the mitigation of mitochondrial dysfunction.

### 3.6. Catalpol Activated Keap1/Nrf2/ARE Pathway in ARPE Cells under Oxidative Stress

**Figure 7:**
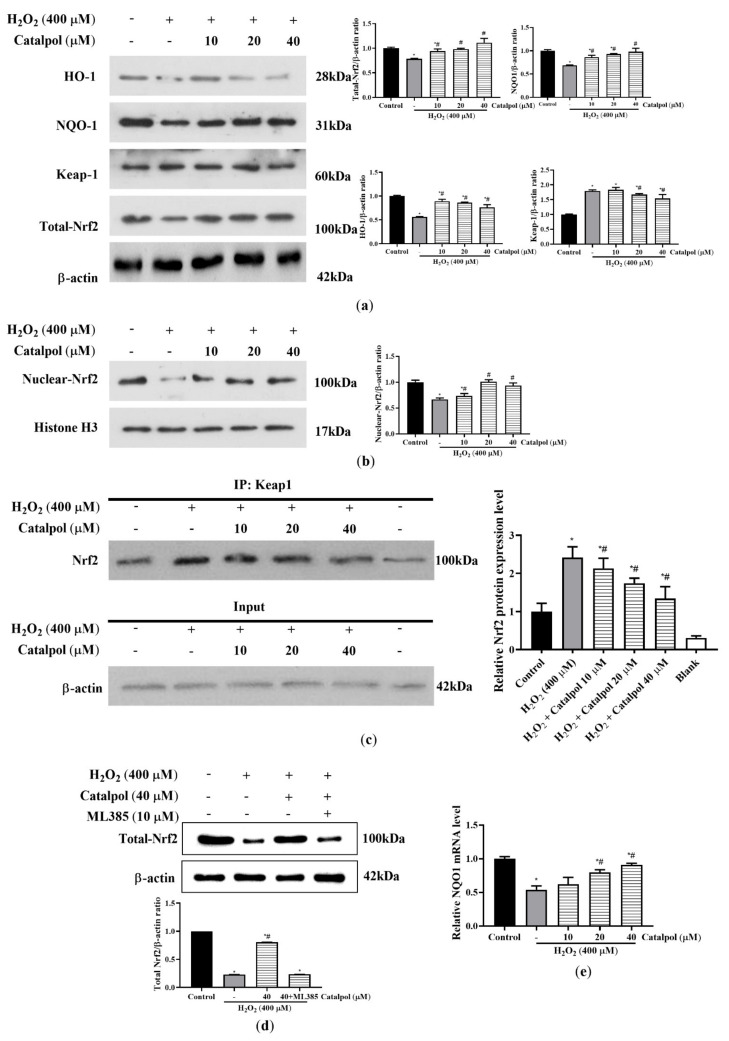
Catalpol activated Keap1/Nrf2/ARE pathway in H_2_O_2_-treated RPE cells. (**a** and **b**) ARPE-19 cells were pretreated with or without catalpol (10, 20 and 40 μM) for 24 h, and then treated with 400 μM H_2_O_2_ for 6 h. The expressions of HO-1, NQO1, Keap1, Total-Nrf2 and Nuclear-Nrf2 were measured with Western blot assay. Histone H3 was used as an internal control for nucleoprotein, while β-actin was used as an internal control for total protein. (**c**) The effect of catalpol on the formation of Keap1/Nrf2 complex was determined using co-immunoprecipitation assay. (**d**) Treatment with ML385, a Nrf2 inhibitor, blocked the antioxidant protective effects of catalpol on increases in the expression of Nrf2 protein in H_2_O_2_-treated ARPE-19 cells. (**e**) Changes in gene expression of NQO1 were assayed with qRT-PCR. Data are presented as mean ± S.D. of three independent experiments (* *p* < 0.05 vs. control group, # *p* < 0.05 vs. H_2_O_2_ (400 μM)-treated group).

The activation of the transcription factor Nrf2 up-regulates the expression of its downstream antioxidant enzymes such as HO-1 and NQO1. Thus, Nrf2 plays a central role in the cellular oxidative stress defense system [37]. We determined whether the regulation of Nrf2/ARE antioxidant system was involved in the cytoprotective effect of catalpol on ARPE-19 cells under H_2_O_2_-mediated oxidative stress. As shown in Figure 7a,b, H_2_O_2_ treatment significantly inhibited the protein expressions of total Nrf2, nuclear Nrf2, and HO-1 and also increased the protein levels of Keap1 in the cytoplasm. Furthermore, the NQO1 protein and mRNA levels were markedly decreased in H_2_O_2_-treated ARPE-19 cells when compared with the control group (Figure 7a,b and e). These changes between the control group and the H_2_O_2_-treated group were significantly reversed by catalpol pretreatment. Under unstimulated conditions, Nrf2 in the cytoplasm is rapidly ubiquitinated and degraded after binding with Keap1, resulting in inhibition of its activity and nuclear translocation [13,14]. The results in Figure 7c show that catalpol pretreatment promoted the disassociation of Nrf2 from Keap1 in the cytoplasm, thereby stimulating Nrf2 protein nuclear translocation and activating the downstream antioxidant enzymes. Moreover, the Nrf2 inhibitor ML385 (at a dose of 10 μM) blocked a catalpol (40 μM)-mediated increase in the expression of total-Nrf2 protein in H_2_O_2_-treated ARPE-19 cells (Figure 7d). These results suggest that the activation of the Keap1/Nrf2/ARE antioxidant pathway may be one of the crucial mechanisms by which catalpol protected ARPE-19 cells from oxidative stress.

## 4. Discussion

Our study suggest that the mechanism involved in the protective effect of catalpol against oxidative stress-induced cell death in ARPE-19 cells may be related to the activation of the Keap1/Nrf2/ARE antioxidant pathway. The results of this study showed that: (1) the cytotoxic effect of H_2_O_2_ on ARPE-19 cells was attenuated in a dose-dependent manner by catalpol pretreatment; (2) catalpol pretreatment restored redox balance in oxidatively damaged cells, which was manifested in reduced levels of ROS and MDA, and increased GSH-Px, CAT, SOD2,SOD, and GSH levels; (3) catalpol inhibited H_2_O_2_-induced apoptosis in ARPE-19 cells by inactivation of the intrinsic and extrinsic apoptotic pathways; (4) within the effective dose range, catalpol enhanced the expressions of Nrf2 and its downstream antioxidant enzymes (HO-1 and NQO1), and (5) pretreatment with catalpol counteracted oxidative stress-mediated cell cycle arrest in ARPE-19 cells.

Previous studies have shown that oxidative stress-mediated apoptosis is one of the mechanisms involved in RPE cell death [10,11]. Exogenous H_2_O_2_ eventually leads to the formation and accumulation of intracellular ROS through simulation of endogenous ROS signaling pathways, thereby suppressing cell growth and triggering mitochondrial-dependent apoptosis [38]. Under oxidative stress, overproduction of intracellular ROS induces mitochondrial dysfunction and increases mitochondrial membrane permeability, leading to the depolarization of mitochondrial membrane potential and the release of cytochrome *c* from mitochondria into the cytoplasm [18,19]. Cytochrome *c*, a key pro-apoptotic protein in intrinsic apoptotic pathways, is also strictly regulated by Bcl-2 family proteins such as anti-apoptotic proteins Bcl-2 and Bcl-XL and pro-apoptotic proteins Bax and Bak [39]. In the cytoplasm, cytochrome *c* activates the downstream caspase-9 and caspase-3 proteins [40]. Caspase-3 is a crucial effector of the execution phase of apoptosis. Its activation leads to the cleavage of the DNA repair enzyme PARP, which triggers irreversible apoptosis [41]. Moreover, capase-8 is an effector of Fas (death receptor protein), whose activation directly activates capase-3, thereby initiating the extrinsic apoptotic pathway [42]. In this study, we first found that catalpol enhanced the survival of ARPE-19 cells and inhibited the increase in apoptotic cells under oxidative stress, suggesting that catalpol is an effective cell protective agent. Further results showed that catalpol significantly inhibited the expression levels of cleaved caspase-9, caspase-3, caspase-8, PARP, Fas, and Bax/Bcl-2 ratios when compared with the H_2_O_2_-treated group, while preventing the release of cytochrome *c*. These results indicate that catalpol protected ARPE-19 cells from oxidative stress by dampening the activation of caspase-dependent mitochondrial and Fas death receptor-mediated apoptotic pathways.

Oxidative stress is known to be important in the pathogenesis of AMD and other degenerative diseases [6,8]. Oxidative stress-mediated overproduction of intracellular ROS disrupts the Bax/Bcl-2 balance and mitochondrial membrane depolarization, which together stimulate the release of cytochrome *c* and trigger mitochondrial-dependent cell apoptosis [43]. In this study, it was observed that the levels of ROS and the Bax/Bcl-2 ratio were significantly increased in ARPE-19 cells stimulated with 400 μM H_2_O_2_, while MMP was significantly decreased. These changes were significantly reversed by catalpol pretreatment. Moreover, catalpol pretreatment significantly reduced the production of MDA in H_2_O_2_-treated ARPE-19 cells, indicating that it mitigated the degree of oxidative damage in cells. Under normal physiological conditions, there is a dynamic balance between levels of intracellular ROS and antioxidants levels; H_2_O_2_ exposure stimulates excessive production of ROS, which leads to disruption of redox balance and oxidative stress-induced damage. The cellular antioxidant defense system involves two modes of action: (1) direct removal of ROS and (2) enhancement of production of natural antioxidants such as GSH and SOD, both of which attenuate oxidative stress-induced damage [44]. It was found that pretreatment with catalpol resulted in marked activation of endogenous antioxidant defense system, including increased levels of SOD and GSH in H_2_O_2_-treated ARPE-19 cells. Moreover, also it was observed that NAC pretreatment effectively blocked H_2_O_2_-induced ARPE-19 cell apoptosis. In general, the results suggest that catalpol may protect ARPE-19 cells from oxidative damage by inhibiting the generation of intracellular ROS, which are involved in the activation of intrinsic apoptotic pathways as upstream effectors.

The transcription factor Nrf2 is involved in regulating cell survival and maintaining redox homeostasis by activating ARE-mediated expressions of antioxidant and phase II detoxification enzymes [45]. Specifically, oxidative stress stimulates the dissociation of Nrf2 from Keap1 and its subsequent transfer to the nucleus. Activated Nrf2 interacts with downstream AREs to initiate transcriptional induction of various antioxidants such as HO-1, NQO1, and SOD [14,15,16]. Heme oxygenase-1 (HO-1), an important antioxidant enzyme in cellular antioxidant defense system, indirectly clears free radicals and repairs DNA damage mediated by oxidative stress [46]. The enzyme NQO1 is involved in phase II detoxification, and it is a ubiquitous enzyme in eukaryotic cells. It prevents the redox reaction of quinone and the formation of reactive oxygen species, and protects the cell from oxidative stress caused by various metabolisms [47,48]. However, some studies have shown that nuclear transfer of Nrf2 still depends on the intensity of oxidative stress, and Keap1 still regulates the redox sensitivity of Nrf2 by controlling the availability of free Nrf2 protein. High-intensity oxidative stress-mediated injury induces generation of a large amount of reactive oxygen species, changes the potential sensitive redox capacity of Keap1, and inhibits the nuclear translocation and accumulation of Nrf2 [20,49,50,51,52]. In this study, the data obtained strongly suggest that catalpol exerted significant antioxidant protection against H_2_O_2_-induced oxidative stress ARPE-19 cells by markedly promoting the accumulation of Nrf2 and its nuclear translocation. In addition, catalpol pretreatment significantly increased the expression levels of antioxidant enzymes HO-1 and NQO1 in ARPE-19 cells. Furthermore, it was confirmed through co-immunoprecipitation experiments that catalpol pretreatment decreased the formation of the Keap1/Nrf2 complex in ARPE-19 cells. We speculate that catalpol pretreatment activates intracellular antioxidant defense mechanisms under oxidative stress and, in particular, may disrupt the cellular processes involved in the formation of the Keap1/Nrf2 complex, thereby promoting Nrf2 translocation. Interestingly, we effectively blocked catalpol-mediated reduction in apoptosis and ROS in H_2_O_2_-treated ARPE-19 cells by pretreatment with ML385 (Figure 2d and Figure 5c). Therefore, these results demonstrate that the activation of the Keap1/Nrf2/ARE antioxidant pathway is one of the critical defense mechanisms by which catalpol exerts its antioxidant effect.

The tumor suppressor p53 is a pivotal nuclear transcription factor that is activated by various cellular stress reactions such as oxidative stress and DNA damage and accumulates in the nucleus, thereby playing an important role in regulation of cell cycle and induction of irreversible apoptosis [53]. The cyclin-dependent kinase inhibitor (CDKI) p21 is a downstream effector of p53, and its activation under oxidative stress indicates that p53-dependent cell cycle arrest is completely triggered [54]. The interaction amongst cyclins, cyclin-dependent kinases (CDK), and CDKI is involved in the regulation of cell cycle. Previous studies have shown that p21 binds to the cyclin-CDK complex, including cyclin E, cyclin A/CDK2, and cyclin D/CDK4, thereby suppressing their kinase activities and eventually inducing cell cycle arrest [55]. In the present study, we found that catalpol pretreatment significantly reduced H_2_O_2_-induced G0/G1 phase cell cycle arrest in ARPE-19 cells. The results of WB further showed that catalpol pretreatment effectively inhibited the protein expression levels of p-p53 and p21, while it increased the expressions of cyclin E, cyclin A, and CDK2. These results indicate that catalpol alleviated the H_2_O_2_-induced cell cycle arrest of ARPE-19 cells by inhibiting the p53/p21 signaling pathway, thereby exerting a cytoprotective effect. In addition, the activation of p53 was demonstrated to stimulate the transcriptional translation of the downstream targetBcl-2 gene family such as Bax, Bcl-2, and Bim, indicating that it participated in the initiation of apoptotic signals [56]. Taken altogether, the results of this study suggest that catalpol protects DNA from oxidative damage by inhibiting ROS production, thereby indirectly reversing cell cycle arrest in G0/G1 phase and inactivating the intrinsic apoptotic pathway.

Furthermore, previous studies found that catalpol was rapidly absorbed by oral administration in rats, and the absolute bioavailability of catalpol in the 50 mg/kg group was 66.7%, indicating its potential for oral administration [57,58]. Wu et al. showed that catalpol may reduce blood glucose and serum inflammatory factor levels in diabetic mice by inhibiting the AGE/RAGE/NF-κB signaling pathway, thus improving retinopathy in diabetic mice [59]. These results provide reference and feasibility for the in vivo study of catalpol in the treatment of AMD. Although our current study only found the antioxidant protective effect of catalpol on ARPE-19 cells, in future studies, we hope to achieve the protective effect of catalpol on RPE cells in an in vivo AMD disease model, so as to further inspire drugs targeting Nrf2 for the treatment of oxidative stress-related degenerative eye diseases.

## 5. Conclusions

The results of this study have demonstrated that catalpol inhibited intracellular ROS overproduction and lipid peroxidation by activating the Keap1/Nrf2/ARE antioxidant pathway, thereby protecting ARPE-19 cells from oxidative stress-induced apoptosis and G0/G1 phase cell cycle arrest. These findings will be useful in the development of novel therapeutic targets and drugs for AMD and oxidative stress-related retinal degenerative diseases.

## Figures and Tables

**Table 1:** All antibodies used in the study are listed below.

Antibodies	Company	Catalogue Number	Concentration
Fas	Abcam	ab82419	1:1000
cytochrome *c*	Abcam	ab90529	1:1000
cleaved caspase 3	Abcam	ab2302	1:1000
cleaved caspase 9	Abcam	ab2324	1:1000
NQO1	Abcam	ab80588	1:1000
p53	Abcam	ab241556	1:1000
Keap1	Abcam	ab118285	1:1000
Bcl-2	Abcam	ab185002	1:1000
Nrf2	Abcam	ab62352	1:1000
CDK2	Abcam	ab32147	1:1000
cyclin A	Abcam	ab33911	1:1000
cyclin E	Abcam	ab181591	1:1000
Bax	Abcam	ab53154	1:1000
β-actin	Abcam	ab8226	1:1000
p21	Abcam	ab188224	1:1000
Histone H3	Abcam	ab1791	1:1000
COX IV	Abcam	ab16056	1:1000
phospho-p53 (Ser15)	Cell Signaling Technology	#9286S	1:1000
cleaved PARP	Cell Signaling Technology	#5625S	1:1000
HO-1	Cell Signaling Technology	#86806S	1:1000
cleaved caspase 8	Cell Signaling Technology	#9496S	1:1000

## Data Availability

Not applicable.
